# The safety and tolerability of alkaloids from *Alstonia scholaris* leaves in healthy Chinese volunteers: a single-centre, randomized, double-blind, placebo-controlled phase I clinical trial

**DOI:** 10.1080/13880209.2021.1893349

**Published:** 2021-04-26

**Authors:** Zhong-Ping Gou, Yun-Li Zhao, Lin-Ling Zou, Ying Wang, Shi-Qing Shu, Xiao-Hong Zhu, Li Zheng, Qi Shen, Zhu Luo, Jia Miao, Yong-Sheng Wang, Xiao-Dong Luo, Ping Feng

**Affiliations:** a Institute of Drug Clinical Trials, West China Hospital, Sichuan University, Chengdu, People’s Republic of China; b State Key Laboratory of Phytochemistry and Plant Resources in West China, Kunming Institute of Botany, Chinese Academy of Sciences, Kunming, People’s Republic of China; c Key Laboratory of Medicinal Chemistry for Natural Resource, Ministry of Education; Yunnan Provincial Center for Research & Development of Natural Products; School of Chemical Science and Technology, Yunnan University, Kunming, People's Republic of China

**Keywords:** Clinical safety, healthy subject, dose-escalation study

## Abstract

**Context:**

Capsule of alkaloids from the leaf of *Alstonia scholaris* (L.) R.Br. (Apocynaceae) (CALAS) is a new investigational botanical drug (No. 2011L01436) for bronchitis, post-infectious cough and asthma.

**Objective:**

To observe the clinical safety and tolerability of CALAS.

**Materials and methods:**

Subjects were assigned to eight cohorts, and each received randomly CALAS or placebo in one of single ascending dose (SAD) of 8, 40, 120, 240, 360, 480, or in one of multiple ascending dose (MAD) of 40 or 120 mg, three times daily for 7 days. Each cohort contained two placebo subjects.

**Results:**

Sixty-two enrolled volunteers completed the study and no serious adverse events and clinically significant changes in vital signs, electrocardiography, and upper abdominal Doppler ultrasonography were observed. The ratios of treatment-emergent adverse events (TEAEs) were reported in 11/46 (23.91%) of CALAS groups and 3/16 (18.75%) of the placebo group (*p* > 0.05), respectively, based on the results of SAD and MAD. All TEAEs were mild, transient, and disappeared without any intervention. The TEAEs possibly related to CALAS treatment were as followings: hiccups (4/46: 8%), dry mouth and nausea (3/46: 6%), increased sleep (2/46: 4%), abdominal distension (1/46: 2%), bilirubin elevated (1/46: 2%).

**Discussion and conclusions:**

CALAS is safe and well-tolerated with no unexpected or clinically relevant safety concerns up to a single dose of 360 mg and three times daily for 7 days up to 120 mg in healthy Chinese volunteers, supporting further Phase II studies.

## Introduction

*Alstonia scholaris* (L.) R.Br. (Apocynaceae), a tropical evergreen tree as folk medicine, is widely distributed in Africa and Asia (Li et al. 1996). The different parts of the plant exhibited diverse bioactivities (Liu et al. 2015), and the remarkable difference of the alkaloidal patterns of *A. scholaris* in different ecological environments was revealed (Yamauchi et al. 1990; Zhang et al. 2014). Moreover, we found that the ecological environment might change plant secondary metabolites significantly (Liu et al. 2013).

In the Dai Ethnic regions (Yunnan Province, China), the *A. scholaris* leaves are considered as a therapy for respiratory disease including those of whooping cough, chronic bronchitis, chronic obstructive pulmonary disease, asthma, etc. At present, the leaf crude water extract is prepared as a tablet or granule and sold as therapeutic drugs for the treatment of tracheitis and the common cold. However, the bioactive compounds of *A. scholaris* for the treatment of respiratory diseases were unrevealed before our research. Our group has extensively studied the characteristics, and the differences in constituents, of different parts of *A. scholaris* (Zhou et al. 2005; Cai et al. 2007, 2010, 2011; Cai, Liu, et al. 2008; Cai, Tan, et al. 2008; Du et al. 2007; Feng et al. 2008, 2009; Xu et al. 2009; Liu et al. 2013; Yang, Qin, et al. 2014; Yang, Luo, et al. 2014; Yang, Yang, et al. 2014; Zhang et al. 2014; Yang et al. 2015; Qin, Zhao, Lunga, et al. 2015; Qin, Zhao, Song, et al. 2015; Chen et al. 2016; Pan et al. 2016). Among the compounds isolated, eight monoterpenoid indole alkaloids from *A. scholaris* have been selected as ‘hot off the press’, as specified in *Natural Products Report* (Hill and Sutherland 2007, 2008, 2011, 2014, 2015, 2016a, 2016b). Moreover, 16 *Alstonia* alkaloids were synthesized by outstanding chemists following our structural identification and bioactivities reported.

Identification of chemical constituents has allowed for further characterization of the activities of indole alkaloids by our team. Shang, Cai, Feng, et al. (2010) reported that the alkaloids fraction from leaf of *A. scholaris* ameliorated edoema, inflammation, and pain through inhibiting the inflammatory mediators (COX-1, COX-2 and 5-LOX) and three indole alkaloids (picrinine, vallesamine and scholaricine) were the principal active components. Meanwhile, the anti-tussive, anti-asthmatic and expectorant effects of alkaloids fraction and three main alkaloids were demonstrated *in vivo* (Shang, Cai, Zhao, et al. 2010). Furthermore, the aforementioned three compounds were identified as potential NF-κB inhibitors using a dual-luciferase reporter assay (Hou, Cao, Wang, et al. 2012) and β_2_AR agonists through a relaxant test on guinea pig tracheal muscles (Hou, Cao, Dong, et al. 2012). In an airway inflammation model of rats, the indole alkaloids extract of *A. scholaris* leaves had a protective effect against airway inflammation and damage induced by lipopolysaccharide (Zhao et al. 2016) and exhibited remarkable antiasthmatic effect through downregulating eosinophils percentage of broncho-alveolar lavage fluid in an ovalbumin-provoked airways allergic asthma model (Zhao et al. 2017). Indole alkaloids of *A. scholaris* also exerted protection against LPS-induced post-infectious cough in mice by decreasing *neutrophils* percentage and C-reactive protein expression (Zhao et al. 2018) and against emphysema (Zhao, Yang, et al. 2020) and pulmonary fibrosis (Zhao, Pu, et al. 2021). Additionally, the antibacterial and anti-viral effect was demonstrated (Zhao, Gou, et al. 2021). These findings led to the hypothesis that indole alkaloids from *A. scholaris* could be used as a novel natural treatment for lung diseases, and may provide lasting benefits to individuals with asthma, post-infection cough, and acute tracheal bronchitis. We further investigated the metabolic characteristics of indole alkaloids using a fully-validated LC-MS/MS method. The time course of plasma concentration in rats fitted an open two-compartment model after intragastrical administration at doses of 10, 25 and 50 mg/kg. Scholaricine, 19-epi-scholaricine were metabolized more rapidly than vallesamine and picrinine (Zhao et al. 2017). In addition, the metabolic pathways mainly focussed on hydroxylation and glucuronidation reactions, a total of 33, 40, and 38 compounds were characterized in plasma, urine and faeces, respectively (Cao et al. 2016).

In terms of preclinical safety evaluation of *A. scholaris* leaves, we performed a systematic toxicological assessment to provide the foundation for a possible comprehensive traditional clinical study. In acute toxicity tests, the toxic symptoms of mice primarily manifested as prone position, shortness of breath, wheezing, and convulsion were observed. The half-lethal dose in mice was 5.48 g/kg/bw, almost 2740 times the clinical dose in humans, and the maximal tolerance dose was 2.2 g/kg/bw (Zhao, Su, et al. 2020). After a single dose (4 g/kg/bw) to a dog, a number of transient symptoms, such as unsteady gait, drooling, emesis, and reddening of peri-oral mucosa, were observed, but no treatment-related mortality (Zhao et al. 2020b). In the chronic toxicity of rats for 17 weeks, the indole alkaloid treated rats did not die and showed no adverse effects or dose-dependent changes in weight or food and water consumption at doses of 50, 100 or 300 mg/kg/bw, despite fluctuations in hematological and biochemical parameters compared with historical data. Furthermore, both gross and histopathological observations revealed no abnormalities in any organ. With daily oral administration to rats, the non-observed-adverse-effect-level was 100 mg/kg/bw (Zhao, Su, et al. 2020). In the chronic toxicity study of dogs with a range of doses of indole alkaloids (20, 60 and 120 mg/kg/bw), there were no toxic symptoms except for emesis and drooling in the majority of animals in the 120 mg/kg/bw treatment group (Zhao et al. 2020b). Moreover, indole alkaloids with or without liver S9-induced metabolic activation showed no genotoxicity in the Ames test, mammalian chromosomal aberration test, or in the micronucleus test, and any adverse effects in any of the nerve, respiratory and cardiovascular systems were not induced, as determined using the safety pharmacology core battery (Zhao et al. 2020a). These results met the requirements for regulatory safety submission as defined by the China Food and Drug Administration (CFDA), and supported a clinical trial application.

Base on the above findings, the capsule of alkaloids from the leaf of *A. scholaris* (CALAS) (No. 2011L01436), and the defined mixture of alkaloids (No. 2011L01665) were developed into a new botanical drug, further approved for phase I/II clinical trials by CFDA. Pharmacokinetics evaluation in healthy Chinese volunteers demonstrated CALAS had a high blood concentration, 19-epischolaricine, vallesamine and pecrinine matched the linear pharmacokinetic characteristics, but scholaricine conformed to the characteristics of nonlinear pharmacokinetics (Li et al. 2019). Here we report the findings of a single centre, double-blind, randomized, placebo-controlled, dose-escalation phase I clinical trial to determine the safety and tolerance of the CALAS in healthy Chinese volunteers.

## Materials and methods

### Materials

The treatment drug (CALAS) was a capsule containing indole alkaloids as the active ingredient. CALAS (40 mg per capsule, lot number: 20120901, expiry date: 2014-09-01; 8 mg per capsule, lot number: 20121101, expiry date: 2014-11-01) and placebo-controlled capsule (made with starch, lot number: 20120903, expiry date:2014-09-03) were supplied by Kunming Institute of Botany, Chinese Academy of Sciences, and Yunnan Institute of Medical Material (Kunming, China). All investigational products were kept in a secure, limited-access storage area under the recommended storage conditions. The capsule preparation was carried out at a manufacturing site with GMP (Good Manufacturing Practices) certification approved by CFDA. In brief, dried and powdered leaves of *A. scholaris* were extracted with 90% EtOH under reflux conditions (3 h × 4) and the solvent evaporated *in vacuo* to obtain ethanol extract. Next, ethanol extract was dissolved in 0.3% aqueous HCl solution and filtered, and the residue was identified as a non-alkaloid fraction referring to previous reports (Shang, Cai, Feng, et al. 2010).

### Dose determination

A single ascending-dose study was designed to investigate the safety and tolerability of single ascending-doses of oral CALAS. According to the Guidelines for the Clinical Research of Chinese Medicine New Drugs (China Food and Drug Administration 2015) and ICH E1-E10 guidelines (International Conference on Harmonization 2002), the starting dose is ∼1 to 2% of the minimum effective dose (China Food and Drug Administration 2015), which is 10 mg/kg in mice. The bodyweight of humans is known to be 60 kg. The theoretical dose was calculated as follows:

①10 mg/kg/bw×1%×60 kg/person=6mg/person

②10 mg/kg/bw×2%×60 kg/person=12mg/person

Thereby, the initial dose of SAD started with 8 mg/person given that the finished product specifications for CALAS is 8 mg/capsule and 40 mg/capsule.

According to the recommendations for guiding principles (China Food and Drug Administration 2015), the maximum dose was 10% of the preclinical dose that caused toxic symptoms or reversible damage to organs in chronic toxicity tests of animals. In our preclinical studies, the slightly toxic responses were observed at the dose of 300 (Zhao, Su, et al. 2020) and 120 mg/kg (Zhao et al. 2020b) in 17-week good laboratory practice (GLP) toxicity pivotal studies of rats and dogs, respectively. The maximum theoretical dose was calculated as follows:

①300 mg/kg/bw×10%×60 kg/person= 1800 mg/person

②120 mg/kg/bw×10%×60 kg/person = 720 mg/person

Thereby, the maximum dose of SAD was 720 mg/person (18 capsules) considering the finished product specifications and clinical operability.

The dose-escalation protocol was determined by modified Fibonacci sequence (Penel and Kramar 2012), that is, six dose level were arranged from the initial dose (8 mg) to the maximum dose (720 mg) including 40 mg (1 capsule/person), 120 mg (3 capsules/person), 240 mg (6 capsules/person), 360 mg (9 capsules/person), 480 mg (12 capsules/person), 600 mg (15 capsules/person).

Multiple ascending-dose study was designed to investigate the tolerability and safety of multiple ascending doses of oral CALAS. The MAD arms were not carried out until after the end of the SAD arms. The upper limit in the MAD arm was selected based on a lower dose than the maximum tolerated dose in SAD arms. The alternative dose groups contain 120 mg/day (1 capsules per time,); 240 mg/day (2 capsules per time); 360 mg/day (3 capsules per time); 480 mg/day (4 capsules per time); 600 mg/day (5 capsules per time); 720 mg/day (6 capsules per time). Each subject received an oral dose of CALAS or placebo three times daily for 7 days. And the specific time of administration was 8:00 AM, 14:00 PM and 20:00 PM.

### Study design

**Figure 1.**
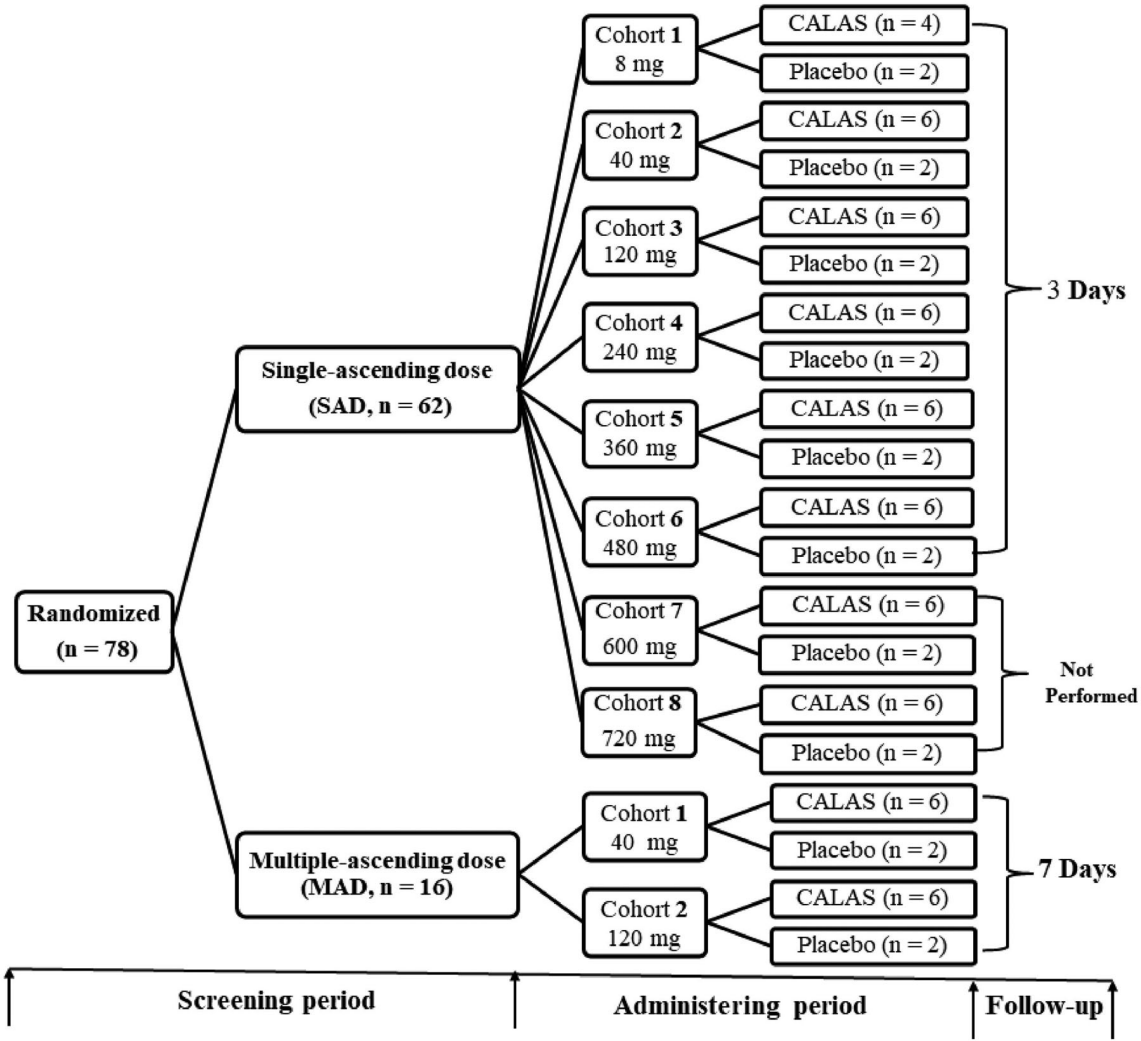
Study flow chart. SAD: Single ascending-dose, once daily; MAD: Multiple ascending-dose, three times daily; CALAS: Capsule of alkaloids from leaf of *A. scholaris*. The further tests of cohort 7 (600 mg) and 8 (720 mg) cohorts in SAD study were not conducted due to the occurrence of adverse reactions in cohort 6 (480 mg).

This was a two-stage, single-centre, double-blinded, randomized, placebo-controlled, dose-escalation study of CALAS in healthy Chinese volunteers (Approval No. 2012-clinical trial-076). The two stages comprised SAD and MAD study shown in the flow chart (Figure 1). In SAD part of the study, 62 subjects were divided into 8 groups; and 16 subjects divided into 2 groups in the MAD study, respectively, each receiving CALAS or placebo in a following single doses of 8, 40, 120, 240, 360, 480, 600, 720 mg, or in one of the multiple doses of 40 or 120 mg, three times daily for seven days. Each cohort comprised eight participants (six in active drug and two in placebo) except that 8 mg cohort contained 4 actives and 2 placebo subjects, the multiple-dose study was performed after the single-dose study. Placebo subjects received capsules containing inactive starch with the same shape, size and smell as CALAS but given merely to satisfy a volunteer who supposed it to be a medicine. At the end of the experiment, subjects in the placebo group in each cohort were pooled as the control group. 8 mg cohort of SAD was dosed first before the followed seven cohorts. Upon review of all available safety data for at least six subjects in the cohort, the safety review committee (SRC) decided to escalate to the next cohort. Namely, after the end of the previous dosing observation, as there were no safety issues from the previous group, the next cohort was dosed.

Subjects in SAD cohorts were admitted to Phase I Clinic Research Unit (PCRU I) ∼24 h (day 0) prior to study drug administration. Subjects remained under clinical supervision for 24 h after administration and kept follow-up visits for 3 days after hospital discharge. The procedures and observation indexes for SAD were shown in Table S1.

Similarly, subjects in the MAD cohort were admitted to PCRU I ∼24 h (day 0) prior to study drug administration. Subjects were hospitalized under clinical supervision for 7 days and kept follow-up observations for 7 days after hospital discharge. The procedures and observation indexes for MAD were shown in Table S2.

### Randomization and masking

Within each dose group, eligible participants were randomly assigned in a 3:1 ratio to CALAS or placebo on day 0 (baseline) except cohort 1 of SAD (2:1). The randomization list was generated using a pseudorandom number generator and a supplied seed number. The randomization list was provided in advance to an unmasked biostatistician at the trial site who was responsible for the blinded drug preparation only. Both active drug and placebo were blue-white capsules. Participants and care providers were kept masked to treatment.

### Participants

The clinical trial was conducted in accordance with the World Medical Association Declaration of Helsinki (2013), the Guideline for Good Clinical Practice (China Food and Drug Administration 2003), and local laws and regulations. The trial was reviewed and approved by the Independent Ethics Committee of West China Hospital of Sichuan University and registered on http://www.chictr.org.cn/showproj.aspx?proj=61093, number ChiCTR2000038081. All subjects provided written informed consents after sufficiently informed about the study before any trial-related procedures were conducted. Subjects were evaluated for eligibility within 2 weeks before dosing, on medical history, vital signs, physical examination, clinical laboratory tests, and electrocardiograph (ECG) recording. Further inclusion criteria were that participants should be healthy male and female (male/female: half/half), non-smokers, and ranging in age from 18 to 45 years, with body mass index (BMI) of 19 to 24 kg/m^2^ and minimum body weight of 45 kg for females and 50 kg for males. Additionally, eligible female subjects were required not to be pregnant, lactating, or of childbearing potential. Other exclusion criteria were as follows: (1) a history of the clinically diagnosed disease in circulatory, respiratory, digestive, urinary, hematological, endocrine/metabolic, neurologic, immunological system, or infectious and psychiatric illness; (2) subjects with seropositive findings for human immunodeficiency virus, hepatitis B virus surface antigen, or anti-hepatitis C virus antibody; (3) subjects with drug allergy history or allergic constitution; (4) subjects who received any other medicine within 2 weeks before dosing; (5) subjects participated in any clinical trial within 1 month before dosing; (6) a history of drug or alcohol abuse, or any condition that could interfere with absorption, distribution, metabolism and excretion of drugs; (7) female subjects who were about to become pregnant or breast-feeding during the trial.

### Safety and tolerability assessments

The primary outcome measures were safety and tolerability. All adverse events (AEs) were monitored and recorded from the treatment start date to the safety follow-up visit 3 days after dosing in the SAD study or 7 days after the last dose in the MAD study for participants. Treatment-emergent adverse events (TEAEs) were defined as AEs occurring after the first dose of treatment or AEs that existed before the first dose but increased in severity after the first dose. Adverse events were coded using the Medical Dictionary for Regulatory Activities version 20.1.

Adverse events (AEs) were recorded and classified as *related, probably related, possibly related, possibly unrelated,* or *unrelated* to the study medication. Adverse drug reactions were adverse events judged to be *related, probably related* or *possibly related* to the study medication.

### Other safety and tolerability assessments

Vital signs were performed in both studies. Measurements were taken at screening, admission and 1, 2, 4, 8, 12 and 24 h after administration in SAD studies. In the MAD study, vital signs were monitored at screening, admission, before administration each day of dosing, within 4 h after each dosing, and 24 h after the day 7 administration (day 8).

Physical examinations and 12-lead electrocardiogram (ECG) were performed at screening, admission, before administration (only to MAD study), and 24 h after administration in the present study.

Serum biochemistry and haematology assessments, urine, and stool analysis were performed at screening, admission, and 24 h after administration in the SAD study. In the MAD study, they were performed at screening, admission, before administration, the 4th day (day 4) during administration and 24 h after the day 7 administration (day 8). The haematology assessment included red blood cell count, haemoglobin, haematocrit, mean cell volume, mean cell haemoglobin, mean cell haemoglobin concentration, white blood cell count, differential white blood cell count, and platelet count. Urinalysis included pH, protein, glucose, ketones, bilirubin, occult blood, leukocytes, urobilinogen, nitrites, and specific gravity testing. Urine microscopy included red blood cells, leukocytes, epithelial cells, casts, crystals, and bacteria. Serum biochemistry analysis included alanine amino transferase (ALT), aspartate amino transferase (AST), total bilirubin (TBIL), direct bilirubin (DBIL), indirect bilirubin (IBIL), total protein (TP), albumin (ALB), gamma glutamyl transpeptidase (GGT), alkaline phosphatase (ALP), urea (BUN), creatinine (Cr).

Upper abdominal ultrasonography with colour Doppler analysis was performed only in MAD study at screening and 24 h after the day 7 administration (day 8).

### Statistics analysis

A *p*-value < 0.05 was considered a statistical difference. Statistical analysis for generating data listings, summary tables and associated figures was performed using SAS 9.3 (SAS Institute Inc., Cary, NC, USA). Safety analysis was performed for all subjects who received at least one treatment dose, and safety variables were summarized using descriptive statistics. Data were summarized descriptively in tabular form. The descriptive analysis method was adopted to describe the demographic characteristics, vital signs, and safety indicators in the form of number, mean, standard deviation, maximum, minimum, and median of each group. Count data are described in frequency list, percentage, or constituent ratio. Inter-group differences in continuous variables were analyzed for significance using analysis of variance (ANOVA) followed by the multiple-comparisons test, or *t*-test or Wilcoxon rank test. Differences in categorical variables were assessed for significance using Chi-squared and Fisher’s exact test. Subjects in the placebo group of each cohort were pooled as the control group in the SAD and MAD study, respectively, when statistics of adverse events and adverse drug reactions were performed.

## Results

### Demographics

According to the experiment protocol, 8 cohorts were planned to be tested for the SAD study. Further tests of 600 and 720 mg cohorts were not conducted due to the occurrence of adverse reactions in 480 mg cohort. Thus, a total of 46 subjects participated in the SAD study of CALAS (34 in the test group, 12 in the placebo group) and entered the result analysis without any dropout. Finally, a total of 16 volunteers (12 in the test group, 4 in the placebo group) completed the MAD study containing 40 and 120 mg cohorts, 3 times a day for 7 days based on the consequence of the SAD study, and no dropouts or premature discontinuations were presented.

Baseline characteristics were similar between the placebo and CALAS groups (data not showed). The mean age (±SD) of the subjects was 28.37 ± 3.13 years (range: 21–43 years), and there was a 1:1 gender distribution. Mean (±SD) height and body mass index were 166.27 ± 7.61 cm (range: 150–180 cm), 20.68 ± 1.37 (range: 19.05–23.99). All subjects had no history of past and present illness, special family, allergy, smoking, and drinking, and met the inclusion criteria. No statistical difference was observed in the other indexes except for the significant difference of age in the MAD study (*p* = 0.01, data not showed) and IBIL (*p* = 0.04), AST (*p* = 0.03), TP (*p* = 0.04) in the SAD study using Wilcoxon rank-sum test for inter-group comparison, all which had no effect on the final evaluation judged by the principal investigator.

### Vital signs results

In the SAD study, the temperature (data not showed) of cohort 2 and 4, the respiratory rate of cohort 1 and the systolic blood pressure of cohort 6 were statistically significant compared with those of placebo (*p* < 0.05). Meanwhile, the respiratory rate of cohort 2, the pulse of cohort 2, 4 and 6, and the heart rates of cohorts 2, 4 and 6 at different time points had statistical differences (*p* < 0.05). In the MAD study, the temperature of cohort 2, the pulse and heart rate of cohort 1 were significantly different by comparison with that of placebo (*p* < 0.05); in addition, the pulse and systolic pressure in cohort 1 at different time points were statistical significance (*p* < 0.05). However, these changes were all in the normal range without clinical significance.

### Clinical laboratory results

As shown in Table 1, SAD study, there was a slight increase in the following indicators in the SAD study: RBC and Hb in 2 cases (pre-dosing vs. post-dosing, cohort 2-1: 5.69 vs. 5.92 × 10^12^/L; cohort 5-7: 4.96 vs. 5.33 × 10^12^/L, range, 4.3–5.8 × 10^12^/L), PLT in 4 cases (pre-dosing vs. post-dosing, cohort 2-2, 296 vs. 319 × 10^9^/L; cohort 3-5, 282 vs. 328 × 10^9^/L; cohort 4-4, 268 vs. 319 × 10^9^/L; cohort 5-8, 371 vs. 398 × 10^9^/L, range: 100–300 × 10^9^/L), neutrophils in 1 case (cohort 5-2, pre-dosing vs. post-dosing = 3.60 vs. 6.46 × 10^9^/L, range: 1.8–6.3 × 10^9^/L), and lymphocytes in 1 case (cohort 5-8, pre-dosing vs. post-dosing = 2.07 vs. 3.3 × 10^9^/L, range: 1.1–3.2 × 10^9^/L), all which were not accompanied by any discomfort or abnormal signs and were considered as clinically insignificant. Urinary protein in 7 cases (cohort 2-1, -2, -5; cohort 4-1, -2; cohort 5-2; cohort 6-2) were presented as ‘±’ at post-dosing and considered to be physiologic proteinuria or pseudoproteinuria without clinical significance evaluated by the principal investigator. Likely, urine alkone in 2 cases (cohort 2-5, cohort 3-5) was displayed as ‘+’ and thought to be caused by fasting, which had no clinical significance. Besides, the TBIL and IBIL of one subject labelled as cohort 5-4 increased slightly (pre-dosing vs. post-dosing: TBIL, 20.7 vs. 30.3 μmol/L, range: 5.0–28 μmol/L; IBIL, 15.6 vs. 22.4 μmol/L, range: <20 μmol/L) but was again within the normal range on the following reinspection for 3 days, were regarded to be possibly related to the study drug. In one subject (cohort 5-8), IBIL was elevated slightly (pre-dosing vs. post-dosing = 7.6 vs. 9.6 μmol/L, range: <8.8 μmol/L) and return to normal at the subsequent reinspection without clinical significance. The value of ALT in one subject cataloged as cohort 1-4 was increased to 41 IU/L from 32 IU/L (range: <40 IU/L) at post-dosing but without any discomfort and considered to be nonclinical significance. The TP and ALB contents of three participants (pre-dosing vs. post-dosing, cohort 2-7: TP, 74 vs. 64.7 g/L; ALB, 45.9 vs. 39.2 g/L; cohort 2-8: ALB, 48.4 vs. 39.4 g/L; cohort 6-3: TP, 69.6 vs. 61.6 g/L; ALB, 45.5 vs. 39.7 g/L) were slightly lower than the normal values (TP: 65-85 g/L; ALB: 40–55 g/L) without clinical significance. In 10 subjects (cohort 1-1; cohort 2-3; cohort 3-1, -3, -4; cohort 4-3; cohort 5-3; cohort 6-2, -3, -5), the ALP content was slightly decreased from 46, 42, 53,49, 51, 54, 35, 43, 54, 44 IU/L to 50, 45, 49, 48, 44, 49, 34, 41, 37, 31 IU/L, respectively without clinical significance, actually, most of them at pre-administration were lower than normal value.

**Table 1.** Abnormal laboratory parameters after single ascending-dose administration.

Code	Items	Indices	Range	Pre-dosing	Post-dosing	Clinical significance	Retesting	Relatedness to treatment (decision of blinded adjudication committee)
Cohort 1-1	Liver function	ALP (IU/L)	51–160	46	50	No	Not performed	Unrelated
Cohort 1-2	Routine urinalysis	Urinary protein (g/L)	(−)	(−)	0.1 (±)	No	Not performed	Unrelated
Cohort 1-4	Hematologic analysis	Hb (g/L)	120–160	160	151	No	Not performed	Unrelated
	Liver function	ALT (IU/L)	<40	32	41	No	Not performed	Unrelated
Cohort 2-1	Hematologic analysis	RBC (×10^12^/L)	4.3–5.8	5.69	5.92	No	Not performed	Unrelated
		Hb (g/L)	130–175	172	178	No	Not performed	Unrelated
	Routine urinalysis	Urinary protein (g/L)	(−)	0.1 (±)	0.1 (±)	No	Not performed	Unrelated
Cohort 2-2	Hematologic analysis	PLT (×10^9^/L)	100–300	296	319	No	Not performed	Unrelated
	Routine urinalysis	Urinary protein (g/L)	(−)	(−)	0.1 (±)	No	Not performed	Unrelated
Cohort 2-3	Liver function	ALP (IU/L)	51–160	42	45	No	Not performed	Unrelated
Cohort 2-4	Hematologic analysis	N%	40–75	59.4	38.9	No	Not performed	Unrelated
		L%	20–50	33.4	52.4	No	Not performed	Unrelated
Cohort 2-5	Routine urinalysis	Urinary protein (g/L)	(−)	(−)	0.1 (±)	No	Not performed	Unrelated
		Urine alkone	(−)	(−)	±	No	Not performed	Unrelated
Cohort 2-7	Liver function	TP (g/L)	65–85	74	64.7	No	68.7	Unrelated
		ALB (g/L)	40–55	45.9	39.2	No	43.2	Unrelated
Cohort 2-8	Liver function	ALB (g/L)	40–55	48.4	39.4	No	43.9	Unrelated
Cohort 3-1	Liver function	ALP (IU/L)	51–160	53	49	No	Not performed	Unrelated
Cohort 3-3	Liver function	ALP (IU/L)	51–160	49	48	No	Not performed	Unrelated
Cohort 3-4	Liver function	ALP (IU/L)	51–160	51	44	No	Not performed	Unrelated
Cohort 3-5	Hematologic analysis	PLT (×10^9^/L)	100–300	282	328	No	Not performed	Unrelated
	Routine urinalysis	Urine alkone	(−)	(−)	(±)	No	Not performed	Unrelated
Cohort 4-1	Routine urinalysis	Urinary protein (g/L)	(−)	0.2 (±)	0.2 (±)	No	0.1 (±)	Unrelated
Cohort 4-2	Routine urinalysis	Urinary protein (g/L)	(−)	(−)	0.1 (±)	No	Not performed	Unrelated
Cohort 4-3	Liver function	ALP (IU/L)	51–160	54	49	No	Not performed	Unrelated
Cohort 4-4	Hematologic analysis	PLT (×10^9^/L)	100–300	268	319	No	Not performed	Unrelated
Cohort 4-7	Liver function	TBIL (μmol/L)	5.0–28.0	8.2	3.3	No	Not performed	Unrelated
Cohort 5-2	Hematologic analysis	N (×10^9^/L)	1.8–6.3	3.60	6.46	No	Not performed	Unrelated
	Routine urinalysis	Urinary protein (g/L)	(−)	(−)	0.1 (±)	No	Not performed	Unrelated
Cohort 5-3	Liver function	ALP (IU/L)	51–160	35	34	No	Not performed	Unrelated
Cohort 5-4	Liver function	TBIL (μmol/L)	5.0–28.0	20.7	30.3	Yes	18.7	Possibly related
		IBIL (μmol/L)	< 20	15.6	22.4	Yes	13.0	Possibly related
Cohort 5-7	Hematologic analysis	RBC (×10^12^/L)	3.8–5.1	4.96	5.33	No	Not performed	Unrelated
		Hb (g/L)	115–150	141	152	No	Not performed	Unrelated
Cohort 5-8	Hematologic analysis	PLT (×10^9^/L)	100–300	371	398	No	Not performed	Unrelated
		L (×10^9^/L)	1.1–3.2	2.07	3.33	No	Not performed	Unrelated
	Liver function	IBIL	<8.8	7.6	9.6	No	7.4	Possibly unrelated
Cohort 6-2	Routine urinalysis	Urinary protein (g/L)	(−)	0.1 (±)	0.1 (±)	No	Not performed	Unrelated
	Liver function	ALP (IU/L)	51–160	43	41	No	Not performed	Unrelated
Cohort 6-3	Liver function	TP (g/L)	65–85	69.6	61.6	No	68.6	Unrelated
		ALB (g/L)	40–55	45.5	39.7	No	45.3	Unrelated
		ALP (IU/L)	51–160	54	37	No	52	Unrelated
Cohort 6-5	Liver function	ALP (IU/L)	51–160	44	31	No	Not performed	Unrelated

−: Negative, ±: Doubtful, +: Positive.

‘Cohort number-number’ of code column represented the specific code of participant with abnormal laboratory parameters in each cohort.

Cohort 1: single ascending-dose, 8 mg per time and once daily; Cohort 2: single ascending-dose, 40 mg per time and once daily; Cohort 3: single ascending-dose, 120 mg per time and once daily; Cohort 4: single ascending-dose, 240 mg per time and once daily; Cohort 5: single ascending-dose, 360 mg per time and once daily; Cohort 6: single ascending-dose, 480 mg per time and once daily.

In the MAD study (Table 2), the PLT content of one subject (cohort 1-2) was slightly increased from 305 × 10^9^/L to 323 × 10^9^/L without clinical significance the baseline of it at pre-administration were higher than the normal value (100–300 × 10^9^/L). The percentage of neutrophil (N%) and lymphocyte (L%) in 3 cases (pre-dosing vs. post-dosing, cohort 2-2: N%, 43.5 vs. 34.7; L%, 43.4 vs. 54.2; cohort 2-3: N%, 48.7 vs. 38.5; L%, 45.7 vs. 54.0; cohort 2-7: L%, 71.0 vs. 75.6) deviated from the normal range (N%, 40–70, L%, 20–50), however, the absolute value of white blood cell, neutrophil and lymphocyte were normal and discomfort and abnormal signs were not observed, thus they were considered as nonclinical significance. The urine protein in 5 subjects (cohort 1-1, -4, -5, -6; cohort 2-3) were presented as ‘1+’ or ‘±’ but were again within the normal value or ‘±’, and considered to be physiologic proteinuria or pseudoproteinuria without any clinical implication. A slight increase of urine white blood cells in one participant (cohort 1-8) from 4/HP to 17/HP was displayed, and judged to be related to urine sample contamination. In one subject (cohort 1-4), ALP (pre-dosing: 54 IU/L, post-dosing: 49 IU/L) and GLB (pre-dosing: 21.8 g/L, post-dosing: 19.6 g/L) were reduced slightly and return to normal (ALP: 51–60 IU/L, GLB: 20–40 g/L) at the subsequent reinspection without clinical significance.

**Table 2.** Abnormal laboratory parameters after multiple ascending-dose administration.

Code	Items	Indices	Range	Pre-dosing	Post-dosing	Clinical significance	Retesting	Relatedness to treatment (decision of blinded adjudication committee)
Cohort 1-1	Routine urinalysis	Urinary protein (g/L)	(−)	(−)	0.2 (±)	No	(−)	Unrelated
Cohort 1-2	Hematologic analysis	PLT (×10^9^/L)	100–300	305	323	No	343	Unrelated
		N%	40–75	43.5	34.7	No	43.1	Unrelated
		L%	20–50	43.4	54.2	No	46.0	Unrelated
Cohort 1-3	Hematologic analysis	N%	40–75	48.7	38.5	No	47.8	Unrelated
		L%	20–50	45.7	54.0	No	45.3	Unrelated
Cohort 1-4	Routine urinalysis	Urinary protein (g/L)	(−)	0.1 (±)	0.3 (1+)	No	(−)	Unrelated
	Liver function	GLB (g/L)	20.0–40.0	21.8	19.6	No	20.5	Unrelated
		ALP (IU/L)	51–160	54	49	No	48	Unrelated
Cohort 1-5	Routine urinalysis	Urinary protein (g/L)	(−)	(−)	0.1 (±)	No	Not performed	Unrelated
Cohort 1-6	Routine urinalysis	Urinary protein (g/L)	(−)	(−)	0.1 (±)	No	Not performed	Unrelated
Cohort 1-7	Hematologic analysis	N%	40–75	71.0	75.6	No	Not performed	Unrelated
Cohort 1-8	Routine urinalysis	WBC (/HP)	0–5	4	17	No	1	Unrelated
Cohort 2-3	Routine urinalysis	Urinary protein (g/L)	(−)	0.1 (±)	0.2 (±)	No	0.1 (±)	Unrelated

−: Negative, ±: Doubtful, +: Positive.

‘Cohort number-number’ of code column represented the specific code of participant with abnormal laboratory parameters in each cohort.

Cohort 1: multiple ascending-dose, 40 mg per time and three times daily for 7 days; Cohort 2: multiple ascending-dose, 120 mg per time and three times daily for 7 days.

The differences of TBIL, DBIL and IDBIL in SAD study were statistically significant by comparison with the placebo (*p* < 0.05, data not showed). The differences between pre-dosing and post-dosing in DBIL, AST, TP, ALB and Cr were shown statistically significant (*p* < 0.05, data not showed). The changes of BUN in CALAS in MAD study were statistically significant in contrast to the placebo (*p* < 0.05). The difference between pre-dosing and post-dosing in GGT and RBC was shown statistically significant (*p* < 0.05). Whereas, the mean values of the above indexes were within the normal range at pre- and post-administration, and had no clinical significance. In addition, no changes in ECG parameters and colour Doppler ultrasonography of the upper abdomen were assessed as clinically significant in the SAD and MAD cohorts. Thereby the changes of the aforementioned parameters were considered not related to CALAS administration by the principal investigator.

### Adverse events (AEs)

No serious adverse events occurred in CALAS- and placebo-treated participants. The incidence of treatment-emergent adverse events (TEAEs) was 11 out of 46 (23.91%) in the CALAS groups and 3 out of 16 subjects (18.75%) in the placebo (Tables 3 and 4). TEAEs in CALAS SAD arms including hiccups (240 mg, *n* = 1; 480 mg, *n* = 3), dry mouth (480 mg, *n* = 3), nausea (480 mg, *n* = 3), abdominal distension (240 mg, *n* = 1), increased bilirubin (360 mg, *n* = 1), and those in CALAS MAD arms were increased sleep (120 mg, *n* = 3), dizziness (120 mg, *n* = 1). All the above TEAEs were mild (Table 5), transient, disappeared without any treatment, and possibly related to CALAS, except for a subject in the MAD cohort with increased sleep and dizziness unlikely to be related to CALAS treatment. TEAEs in placebo were loose stools (*n* = 1), dry mouth (*n* = 1) and diarrhoea (*n* = 1), and which were mild, transient, and resolved spontaneously, further judged unlikely to be related to placebo treatment.

**Table 3.** Adverse events profile with a single ascending-dose of CALAS.

TEAEs	Cohort 1		Cohort 2		Cohort 3		Cohort 4		Cohort 5		Cohort 6		Total	
	CALAS	Placebo	CALAS	Placebo	CALAS	Placebo	CALAS	Placebo	CALAS	Placebo	CALAS	Placebo	CALAS	Placebo
*n* (Missing)	4 (0)	2 (0)	6 (0)	2 (0)	6 (0)	2 (0)	6 (0)	2 (0)	6 (0)	2 (0)	6 (0)	2 (0)	34 (0)	12 (0)
Without (%)	4 (100.00)	2 (100.00)	6 (100.00)	2 (100.00)	6 (100.00)	2 (100.00)	5 (83.33)	1 (50.00)	5 (83.33)	2 (100.00)	0 (0.00)	1 (50.00)	26 (76.47)	10 (83.33)
With (%)	0 (0.00)	0 (0.00)	0 (0.00)	0 (0.00)	0 (0.00)	0 (0.00)	1 (16.67)	1 (50.00)	1 (16.67)	0 (0.00)	6 (100.00)	1 (50.00)	8 (23.53)	2 (16.67)
Statistics														
*p-*Value							0.46		1.00		0.25		1.00	

Fisher’s exact test was used.

TEAEs: Treatment-emergent adverse events; *n*: the number of all participants; %: the percentage of participants with or without adverse reactions; Without: the number of cases without adverse events; With: the number of cases with adverse events.

Cohort 1: single ascending-dose, 8 mg per time and once daily; Cohort 2: single ascending-dose, 40 mg per time and once daily; Cohort 3: single ascending-dose, 120 mg per time and once daily; Cohort 4: single ascending-dose, 240 mg per time and once daily; Cohort 5: single ascending-dose, 360 mg per time and once daily; Cohort 6: single ascending-dose, 480 mg per time and once daily.

**Table 4.** Adverse events profile with a multiple ascending-dose of CALAS.

TEAEs	Cohort 1		Cohort 2		Total	
	CALAS	Placebo	CALAS	Placebo	CALAS	Placebo
*n* (Missing)	6 (0)	2 (0)	6 (0)	2 (0)	12 (0)	4 (0)
Without (%)	6 (100.00)	1 (50.00)	3 (50.00)	2 (100.00)	9 (75.00)	3 (75.00)
With (%)	0 (0.00)	1 (50.00)	3 (50.00)	0 (0.00)	3 (25.00)	1 (25.00)
Statistics						
*p-*Value			0.46		1.00	

Fisher’s exact test was used.

TEAEs: treatment-emergent adverse events; *n*: the number of all participants; Without: the number of cases without adverse events; With: the number of cases with adverse events; %: the percentage of participants with or without adverse reactions.

Cohort 1: multiple ascending-dose, 40 mg per time and three times daily for 7 days; Cohort 2: multiple ascending-dose, 120 mg per time and three times daily for 7 days.

**Table 5.** Adverse events with CALAS in grades.

Group	Items	Mild (*n*, %)		Moderate (*n*, %)		Severe (*n*, %)	
		CALAS	Placebo	CALAS	Placebo	CALAS	Placebo
SAD	Abdominal distension	1 (2.94)	0 (0.00)	0 (0.00)	0 (0.00)	0 (0.00)	0 (0.00)
	Hiccups	4 (11.76)	0 (0.00)	0 (0.00)	0 (0.00)	0 (0.00)	0 (0.00)
	Loose stools	0 (0.00)	1 (8.33)	0 (0.00)	0 (0.00)	0 (0.00)	0 (0.00)
	Increased bilirubin	1 (2.94)	0 (0.00)	0 (0.00)	0 (0.00)	0 (0.00)	0 (0.00)
	Dry mouth	3 (8.82)	1 (8.33)	0 (0.00)	0 (0.00)	0 (0.00)	0 (0.00)
	Nausea	3 (8.82)	0 (0.00)	0 (0.00)	0 (0.00)	0 (0.00)	0 (0.00)
MAD	Diarrhoea (%)	0 (0.00)	1 (25.00)	0 (0.00)	0 (0.00)	0 (0.00)	0 (0.00)
	Increased sleep (%)	3 (25.00)	0 (0.00)	0 (0.00)	0 (0.00)	0 (0.00)	0 (0.00)
	Dizziness (%)	1 (8.33)	0 (0.00)	0 (0.00)	0 (0.00)	0 (0.00)	0 (0.00)

SAD: single ascending-dose; MAD: multiple ascending-dose.

The symbols *n* and % represented the participants with adverse reactions, with *n* indicating number and % indicating percentage.

### Adverse drug reactions (ADRs)

The ratio of total ADRs in the CALAS group was 10/46 (21.7%), and all were mild in severity (Tables 6–8). No ADRs were reported in the placebo group during the trial. In SAD arms (Table 6), ADRs distributed in 240, 360, 480 mg, the ratios were 1/6, 1/6, 6/6, respectively, and 2/6 in the 240 mg of MAD arms. The most frequently reported ADRs were hiccups (4/46), and the second was dry mouth and nausea (3/46), the ratios of other ADRs such as increased sleep, abdominal distension and elevated bilirubin were 2/46, 1/46, 1/46, respectively.

**Table 6.** Adverse drug reactions profile with a single ascending-dose of CALAS.

ADRs	Cohort 1		Cohort 2		Cohort 3		Cohort 4		Cohort 5		Cohort 6		Total	
	CALAS	Placebo	CALAS	Placebo	CALAS	Placebo	CALAS	Placebo	CALAS	Placebo	CALAS	Placebo	CALAS	Placebo
*n* (Missing)	4 (0)	2 (0)	6 (0)	2 (0)	6 (0)	2 (0)	6 (0)	2 (0)	6 (0)	2 (0)	6 (0)	2 (0)	34 (0)	12 (0)
Without (%)	4 (100.00)	2 (100.00)	6 (100.00)	2 (100.00)	6 (100.00)	2 (100.00)	5 (83.33)	2 (100.00)	5 (83.33)	2 (100.00)	0 (0.00)	2 (100.00)	26 (76.47)	12 (100.00)
With (%)	0 (0.00)	0 (0.00)	0 (0.00)	0 (0.00)	0 (0.00)	0 (0.00)	1 (16.67)	0 (0.00)	1 (16.67)	0 (0.00)	6 (100.00)	0 (0.00)	8 (23.53)	0 (0.00)
Statistics														
*p*-Value										1.00				

Fisher’s exact test was used.

ADRs: Adverse drug reactions; *n*: the number of all participants; Without: the number of cases without adverse reactions; With: the number of cases with adverse reactions; %: the percentage of participants with or without adverse reactions.

Cohort 1: single ascending-dose, 8 mg per time and once daily; Cohort 2: single ascending-dose, 40 mg per time and once daily; Cohort 3: single ascending-dose, 120 mg per time and once daily; Cohort 4: single ascending-dose, 240 mg per time and once daily; Cohort 5: single ascending-dose, 360 mg per time and once daily; Cohort 6: single ascending-dose, 480 mg per time and once daily.

**Table 7.** Adverse drug reactions profile with a multiple ascending-dose of CALAS.

ADRs	Cohort 1		Cohort 2		Total	
	CALAS	Placebo	CALAS	Placebo	CALAS	Placebo
*n* (Missing)	6 (0)	2 (0)	6 (0)	2 (0)	12 (0)	4 (0)
With (%)	0 (0.00)	0 (0.00)	2 (33.33)	0 (0.00)	2 (16.67)	0 (0.00)
Without (%)	6 (100.00)	2 (100.00)	4 (66.67)	2 (100.00)	10 (83.33)	4 (100.00)
Statistics						
*p*-Value			1.00		1.00	

Fisher’s exact test was used.

ADRs: Adverse drug reactions; *n*: The number of all participants; With: the number of cases with adverse reactions; Without: the number of cases without adverse reactions; %: the percentage of participants with or without adverse reactions.

Cohort 1: multiple ascending-dose, 40 mg per time and three times daily for 7 days; Cohort 2: multiple ascending-dose, 120 mg per time and three times daily for 7 days.

**Table 8.** Adverse drug reactions in grades.

Group	Items	Mild (*n*, %)		Moderate (*n*, %)		Severe (*n*, %)	
		CALAS	Placebo	CALAS	Placebo	CALAS	Placebo
SAD	Hiccups	4 (11.76)	0 (0.00)	0 (0.00)	0 (0.00)	0 (0.00)	0 (0.00)
	Dry mouth	3 (8.82)	1 (8.33)	0 (0.00)	0 (0.00)	0 (0.00)	0 (0.00)
	Nausea	3 (8.82)	0 (0.00)	0 (0.00)	0 (0.00)	0 (0.00)	0 (0.00)
	Abdominal distension	1 (2.94)	0 (0.00)	0 (0.00)	0 (0.00)	0 (0.00)	0 (0.00)
	Increased bilirubin	1 (2.94)	0 (0.00)	0 (0.00)	0 (0.00)	0 (0.00)	0 (0.00)
MAD	Increased sleep (%)	2 (16.67)	0 (0.00)	0 (0.00)	0 (0.00)	0 (0.00)	0 (0.00)

SAD: single ascending-dose; MAD: multiple ascending-dose.

The symbols *n* and % stand for the participants with adverse reactions, with *n* indicating number and % indicating percentage.

### Maximum tolerated dose (MTD)

In the SAD study, half of the subjects experienced mild adverse drug reactions when the dose ascended to 480 mg (12 capsules), and that met the criteria for termination of the trial reviewed by the clinical adjudication committee. Therefore, the lower dose – 360 mg (9 capsules) – was determined to be the maximum tolerated dose of this product for a single dose oral administration.

## Discussion

This is phase I, single-centre, randomized, double-blind, placebo-controlled study of CALAS in healthy adult volunteers. In this study, vital signs, clinical symptoms, biochemical parameters, electrocardiogram and upper abdominal ultrasonography were measured throughout the study to assess the toxicity of CALAS, the finds indicated that CALAS in healthy Chinese subjects with oral administration of single (8, 40, 120, 240, 360, 480 mg) and multiple (40 and 120 mg t.i.d) doses of CALAS were well tolerated and associated with minor side effects in 62 healthy volunteers.

The rate of TEAEs was slightly higher in CALAS subjects than those in placebo subjects (23.91% vs. 18.75%). The TEAEs in CALAS groups were mainly manifested as hiccups, dry mouth, nausea, abdominal distension, increased bilirubin or sleep and those in the placebo group were loose stools, dry mouth and diarrhoea. which were mild, transient and spontaneously resolved. In terms of adverse drug reactions, there were 1, 1, 6 cases, respectively, in single 240, 360, 480 mg dose groups and 2 cases in MAD 120 mg group, indicating that the incidence rate of adverse drug reactions increased along with the dose increasing. The fact that Yang and Zhao (2013), showed CALAS has an antagonistic action against bronchoconstrictor responses on the guinea pig induced by histamine and acetylcholine, suggesting that CALAS might also be antagonizing acetylcholine receptor. Another study evaluating acute and chronic toxicity of CALAS in beagle dogs (Zhao et al. 2020b), showed several transient symptoms, such as unsteady gait, drooling, and emesis. Obviously, similar dyspeptic symptoms were observed both in animals and humans. Accordingly, we postulated that the dry mouth was partially due to the anticholinergic effect of CALAS and dyspeptic symptoms resulted from the bitter powder adsorbing in the capsule shell stimulated the oral mucosa and taste buds. However, the dyspeptic symptoms were not reported in the MAD study, which may be related to the fact that it occurred in high-dose levels in SAD group, and although multiple doses were given, each dose was low and not enough to cause the adverse effects. Nevertheless, the exact mechanism that caused sleep increase in the MAD group was unclear. Whether this product has sedative and hypnotic effects remains to be further clarified in future clinical trials. In addition, our results did not reveal any significant change in anaphylactic allergic reactions but were observed in dogs of acute toxicity tests such as reddening of peri-oral mucosa, which needs to be further elucidated in future clinical trials.

In this study, there were some statistically significant differences in mean values in some vital signs, laboratory indexes between the CALAS group and the placebo group, as well as pre- and post-dosing. However, the mean values of the above indicators were all within the normal range and had no clinical significance. The main reasons for the statistical differences may be as follows: first, each subject has different functional states and physiological fluctuations with time; secondly, there is an intra- and inter-day laboratory analysis error within the allowable range, which makes the measured value fluctuate within the normal range. Moreover, the number of subjects is small and the statistical sample size is insufficient, leading to a statistical bias.

Overall, this is the first phase I clinical trial in healthy adults evaluating safety and tolerance of CALAS, indicating that it was safe and well-tolerated in the range of 8–360 mg in SAD, and the maximum tolerated dose of SAD was 360 mg. Multiple doses of 120 mg per time, 3 times daily, for 7 days were safe and well-tolerated. According to the adverse effects presented by the study, larger samples in further phase II–IV clinical trials should be conducted to evaluate its safety deeply and comprehensively, and may be able to elucidate the side effects in the digestive system, nerve system and liver function further. The study duration was also limited to 7 days in the MAD study, longer durations of administration should be reasonable to identify side effects with long term therapy.

## Conclusions

CALAS is a new investigational botanical drug derived from indole alkaloids extract of *A. scholaris* leaves with the potential to improve outcomes in the acute and chronic respiratory disease when compared to similar preparations on sale. This phase I study demonstrated the safety profile was reassuring, demonstrating only mild, transient adverse effects. Taken together, this favourable safety and tolerance profile in humans, the wealth of preclinical data demonstrating a histologically and functionally therapeutic benefit across different models of respiratory infections, and reliable quality and stability of the product support further development of CALAS for the treatment of patients with respiratory diseases. The recommended dosage regimen for phase II clinical trials is 40–120 mg, 3 times daily.

## Supplementary Material

Supporting_Materials-R2.docx
