# Supplementary material for: The safety and tolerability of alkaloids from Alstonia scholaris leaves in healthy Chinese volunteers: a single-centre, randomized, double-blind, placebo-controlled phase I clinical trial
Source: Pharm Biol. 2021 Apr 26;59(1):482–91. doi: 10.1080/13880209.2021.1893349 (PMC8086589; doi:10.1080/13880209.2021.1893349)
Supplement: Supporting_Materials-R2.docx [file IPHB_A_1893349_SM2570.docx]

**Supporting Materials for**

**The safety and tolerability of alkaloids from *Alstonia schola*ris leaves in healthy Chinese volunteers: A single-center, randomized, double-blind, placebo-controlled phase I clinical trial**

## Table S1. Determination of index and frequency in SAD study

| **Items** | Screening period (Day -15 - 0) | Day 0  (Administration) | 24 hours after drug withdrawal | Follow-up period (Day 1 - 3 after drug withdrawal) |
| --- | --- | --- | --- | --- |
| Informed consent | X |  |  |  |
| Demographic data and medical history | X |  |  |  |
| Smoking and drinking history | X |  |  |  |
| Inclusion/exclusion criteria | X |  |  |  |
| Physical examination | X |  | X |  |
| Vital signs* | X | X | X |  |
| Blood, urine and stool examination | X |  | X |  |
| Liver and kidney function test | X |  | X |  |
| Hepatitis B and anti-HIV antibody tests | X |  |  |  |
| HCG examination * | X |  |  |  |
| ECG examination | X |  | X |  |
| Taking CALAS |  | X |  |  |
| Clinical monitoring |  | X | X |  |
| Drug combination | X | X | X | X |
| Adverse events |  | X | X | X |

HIV: Human Immunodeficiency Virus; HCG: Human Chorionic Gonadotropin: ECG: Electrocardiograph.

*: Vital signs were performed at pre-dosing and post-dosing for 1, 2, 4, 8, 12, 24 hours. Dynamic monitoring of vital signs when necessary. HCG examination is suitable for women of childbearing age.

## Table S2. Determination of indices and frequencies in multiple ascending-dose (MAD) study

| Items | Screening period (Day -15 ~ 0) | Administration  (Day 1 - 7) | | | 24 hours after drug withdrawal (Day 8) | Follow-up period (Day 1 - 7 after drug withdrawal) |
| --- | --- | --- | --- | --- | --- | --- |
| Informed consent | X | Day 1 - 3 | Day 4 | Day 5 - 7 |  |  |
| Demographic data | X |  |  |  |  |  |
| Smoking and drinking history | X |  |  |  |  |  |
| Inclusion/exclusion criteria | X |  |  |  |  |  |
| Physical examination | X |  |  |  | X |  |
| Vital signs | X | X | X | X | X |  |
| Blood, urine and stool examination | X |  | X |  | X |  |
| Liver and kidney function test | X |  | X |  | X |  |
| Hepatitis B and anti-HIV antibody tests | X |  |  |  |  |  |
| HCG examination * | X |  |  |  |  |  |
| ECG examination | X |  |  |  | X |  |
| Color doppler ultrasound examination of upper abdomen | X |  |  |  | X |  |
| Taking CALAS |  | X | X | X |  |  |
| Clinical monitoring |  | X | X | X | X |  |
| Drug combination | X | X | X | X | X | X |
| Adverse events |  | X | X | X | X | X |

HIV: Human Immunodeficiency Virus; HIV: Human Chorionic Gonadotropin: ECG: Electrocardiograph.

*: Vital signs were performed at pre-dosing and 4 h of post-dosing. Dynamic monitoring of vital signs when necessary. HCG examination is suitable for women of childbearing age.
